# Risk Factors of Fall-Related Emergency Department Visits by Fall Location of Older Adults in the US

**DOI:** 10.5811/westjem.2021.2.49307

**Published:** 2021-07-19

**Authors:** Uma Kelekar, Debasree Das Gupta, Jewel Goodman Shepherd, Anupam A. Sule

**Affiliations:** * Marymount University, College of Business, Innovation, Leadership and Technology, Division of Health Care Management, Arlington, Virginia; † Utah State University, Department of Kinesiology and Health Science, Logan, Utah; ‡ University of South Dakota, Beacom School of Business, Vermillion, South Dakota; § St. Joseph Mercy Oakland, Department of Informatics and Outcomes, Pontiac, Michigan

## Abstract

**Introduction:**

Prior evidence indicates that predictors of older adult falls vary by indoor-outdoor location of the falls. While a subset of United States’ studies reports this finding using primary data from a single geographic area, other secondary analyses of falls across the country do not distinguish between the two fall locations. Consequently, evidence at the national level on risk factors specific to indoor vs outdoor falls is lacking.

**Methods:**

Using the 2017 Nationwide Emergency Department Sample (NEDS) data, we conducted a multivariable analysis of fall-related emergency department (ED) visits disaggregated by indoor vs outdoor fall locations of adults 65 years and older (N = 6,720,937) in the US.

**Results:**

Results are compatible with findings from previous primary studies. While women (relative risk [RR] = 1.43, 95% confidence interval [CI], 1.42–1.44) were more likely to report indoor falls, men were more likely to present with an outdoor fall. Visits for indoor falls were highest among those 85 years and older (RR = 2.35, 95% CI, 2.33–2.37) with outdoor fall visits highest among those 84 years and younger. Additionally, the probabilities associated with an indoor fall in the presence of chronic conditions were consistently much higher when compared to an outdoor fall. We also found that residence in metropolitan areas increased the likelihood of an indoor elderly fall compared to higher outdoor fall visits from seniors in non-core rural areas, but both indoor and outdoor fall visits were higher among older adults in higher income ZIP codes.

**Conclusion:**

Our findings highlight the contrasting risk profile for elderly ED patients who report indoor vs outdoor falls when compared to the elderly reporting no falls. In conjunction, we highlight implications from three perspectives: a population health standpoint for EDs working with their primary care and community care colleagues; an ED administrative vantage point; and from an individual emergency clinician’s point of view.

## INTRODUCTION

Older patients represent a quarter of United States (US) emergency department (ED) visits,^1^ and falls are among the most common conditions encountered in EDs.^1^ With the progressive aging of the US population, the number of falls and fall-related ED visits among older adults (≥65 years) is increasing.^2^ Prior studies document the high volume of ED visits for falls,^2^,^3^ the substantial medical costs,^4^ and the health burden^4^ associated with falls among US older adults. Accordingly, Healthy People 2020 aims to reduce fall-related ED visits by 10%,^5^ making fall prevention a priority in public health.^6^

The etiology of older adult falls is complex. Falls may result from an underlying pathology related to chronic conditions^7^–^9^ or may be due to general frailty.^7^ In addition to intrinsic (personal) conditions, the literature^10^,^11^ highlights situational (activity at the time of fall) and extrinsic (environmental) factors as significant drivers of older adult falls. In conjunction, prior US studies^12^,^13^ have distinguished falls by location – indoors vs outdoors – and highlighted that the intrinsic predictors associated with each are different. Despite their significance, the generalizability and reliability of these prior findings^12^–^15^ are limited by the single geographic area, small sample size, and the self-reported data on falls considered in these analyses.

Conversely, a large body of research^2^,^16^ examines characteristics of fall-related ED visits in the US at the national level, but no studies have conducted analysis disaggregated by fall location. Consequently, national trends differentiating indoor from outdoor falls and/or fall-related ED visits among older adults remain unknown. Our goal in this study was to evaluate whether the predictors of fall-related ED visits across the US differed by fall location. Using the 2017 Nationwide Emergency Department Sample (NEDS) data, we examined the role of patient characteristics (gender, age groups, and multiple chronic conditions) after controlling for personal- (insurance) and community-level (location and income) enabling resources.

## METHODS

### Study Design and Setting

We used the 2017 NEDS dataset for our analysis. While national-level statistics on falls in the US arise out of self-reported information (for example, the Behavioral Risk Factor Surveillance System or the National Health Interview Survey), NEDS is the one exception. This dataset has a robust sample size (N = 33 million [unweighted], 145 million [weighted] observations in 2017) and is also the largest, all-payer ED database in the US. It provides national estimates of hospital-based ED visits using a stratified, single-stage cluster sample across 20% of the community, non-rehabilitation hospitals in the US. The NEDS dataset includes information on both patient- and hospital-level characteristics, principal and secondary payers for ED services rendered, and principal diagnosis with up to 35 secondary diagnoses reported using the *International Classification of Diseases, Tenth Revision, Clinical Modification* (ICD-10-CM) codes. Our study was exempt from a review by Marymount University’s institutional review board, and all coauthors completed the Healthcare Cost and Utilization Project data use agreement.

**Population Health Research Capsule**

What do we already know about this issue?

*Personal and environmental predictors of older adult falls, specifically indoor vs outdoor falls, have been explored in prior, small sample studies*.

What was the research question?

*Across the US, do the predictors of falls-related ED visits differ by indoor vs outdoor fall locations of older adults?*

What was the major finding of the study?

*Indoor and outdoor falls varied significantly based on gender, age, urbanity, and chronic health conditions of older adults*.

How does this improve population health?

*Targeted indoor-falls prevention based on contrasting risk profile of indoor/outdoor elderly falls has the potential to address increasing volume of fall-related ED visits in this population*.

### Outcome and Predictor Variables

**Figure 1:**
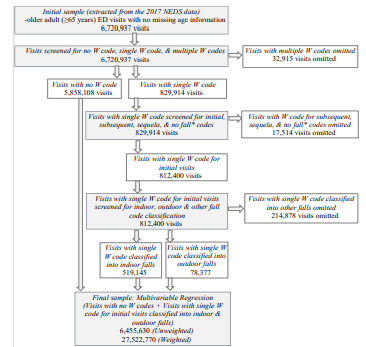
Sample extraction and selection/exclusion criteria using the Nationwide Emergency Department Sample (NEDS) 2017. Note: Data extraction and statistical analysis were conducted by the study authors. *No falls = Jumping/diving, and slipping, tripping, stumbling without falling.

We identified fall-related ED visits (N = 6,720,937) for older adults using ICD-10-CM diagnosis codes for an initial visit (W00-W19) as the sole listed fall diagnosis code across all 35 diagnoses. In Figure 1, we provide a visual representation of the sample extraction and sample selection/exclusion criteria using NEDS 2017. The definition of indoor/outdoor falls in Kelsey et al (2010)^12^ guided how we identified and grouped the W codes into the two fall categories of indoors and outdoors. The W codes we could not assign either as an indoor or an outdoor fall were grouped together into the “other” fall category. Additional details on the W codes and our indoor-outdoor fall classification are provided in Table 1. The unit of our analysis was an ED visit, and the outcome variable was fall-related ED visits for indoor and outdoor falls. We considered the following patient (personal/intrinsic) characteristics: age (age groups), gender, and health status (multiple chronic conditions). Sociodemographic characteristics are consistently identified in the literature as significant predictors of falls,^7^,^8^ falls by location,^10^,^12^–^15^ and fall visits.^2^,^16^ The role of poor health, especially multiple chronic health conditions, is also identified in prior studies^7^–^9^ on older adult falls, and we were particularly interested in examining associations for fall visits disaggregated by fall location. Given that the pattern of chronic conditions significantly predicts the risk of falls^7^–^9^ and increases the likelihood of mortality of older adults^10^,^18^ we aimed to interpret the impact of multiple chronic health conditions. We therefore examined the likelihood of a fall in the presence of a group as well as a set of individual chronic conditions added to our original regression model.

We considered the set of individual chronic conditions identified in the computation of the Charlson Comorbidity Index (CCI).^19^ The CCI is a weighted index that takes into account the seriousness of a set of specific comorbid conditions to predict risk of death following hospitalization.^20^ The CCI is generated based on weights assigned to 17 chronic conditions. The cumulative weights are then grouped into a three-category [0,1,2] Grouped Charlson Comorbidity Index (GRPCI). The concepts of the CCI and GRPCI are used widely to estimate comorbid burden in health services research using large secondary hospital datasets. A list of these comorbid conditions is indicated in Table 1.

Lastly, we also considered the following covariates in our analyses: insurance; and location (rurality/urbanity) and income of patient’s ZIP code. These personal (insurance) and community-level (income and care availability by rurality/urbanity) factors are “enabling resources” that typically influence utilization of health services, including ED services.^21^ Additional details on these explanatory factors (predictors) and the outcome variable are in Table 1.

### Data Analysis

We computed national estimates for all fall categories from which we calculated the rates (per 100,000 older- adult population) of ED fall-related visits across the three age groups: 65–74 years; 75–84 years; and 85 years and above. The population estimates for those 65 years and older for the calculation of these rates were obtained from the US Census Bureau.^22^ We also computed descriptive statistics to summarize the characteristics of fall-related visits by fall locations (indoor and outdoor) across all predictor variables. We conducted both bivariate (chi-square) and multivariable (multinomial logistic regression) analyses to examine heterogeneity, if any, of predictors by fall location of older adult ED visits in the US. We performed all statistical analyses using Stata 15 (StataCorp, College Station, TX). All estimates are weighted unless specified otherwise. We report national estimates and statistically significant findings at *P* ≤ 0.05 unless otherwise noted.

## RESULTS

### National Estimates

We estimated the total volume of ED visits among older adults (≥ 65 years) in 2017 to be about 29 million (28,988,938). Based on 812,400 (unweighted) falls treated in the ED, we estimated about 12.18% (3,529,861 visits) of the total older adult ED visits were fall related. The annual ED charges for these fall visits were $17.3 billion, with an average charge of $5,765 per visit. The average charge for an indoor fall was the highest ($5,820), followed by outdoor falls ($5,730), and “other” falls ($5,511).

### Descriptive Statistics and Rates by Fall Categories

**Figure 2:**
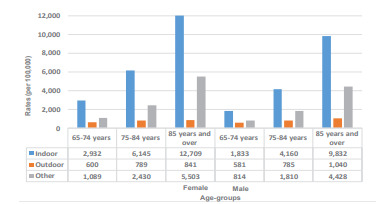
Indoor, outdoor, and other falls stratified by gender and age, NEDS* 2017. Rates of indoor, outdoor and “other” falls by gender and by age categories demonstrating a higher incidence of falls among women, advancing with age (for both gender). Note: We calculated the rate for each fall type by dividing the total number of falls in each age/gender category with the total number of population in that age/gender category. *NEDS, Nationwide Emergency Department Sample.

When compared across the type of fall setting, 64% were indoor (2,247,417), 10% were outdoor (349,632), and the remaining 26% (932,812) were in the “other” setting. Figure 2 depicts the rates of indoor, outdoor, and “other” falls by gender and age categories. Rates for both indoor and outdoor, as well as “other” fall visits, increased sharply across the three age categories for women as well as men. However, this increase for both genders was the starkest for the indoor category (blue bars) with the largest rate increase recorded among the 85 years and older group. When compared across the type of falls, among both men and women, the rate of indoor falls increased almost fivefold among those 85 years and older compared to the 65–74 years group. On the other hand, for outdoor fall-related ED visits, the difference by age groups was less than twice in men and women. Furthermore, for any given age category (except outdoor falls for 85 years and older), the rate of fall-related ED visits was higher in women than men for indoor and outdoor falls. These trends were consistent for the rates of “other” falls.

#### Bivariate Analysis

In Table 2, we list descriptive statistics summarizing total ED visits, and total fall-related ED vists, as well as indoor and outdoor falls by fall predictors of the elderly. The ED visits among older adults were highest in women (56.95%), 65–74 year olds (45.31%), Medicare beneficiaries (87.26%), those living in metro areas (81.0%), and among those in ZIP codes with incomes below $51,000 (55.1%). Similarly, a majority of the falls seen in the EDs were among women (65.20%), older adults 75 and over (66.70%), Medicare beneficiaries (89.5%), in large metro areas (48.91%), and among income groups below $51,000 (51.45%).

The bivariate analysis indicated that the type of falls varied significantly across gender, age group, location, payer, income, and GRPCI (*P* <0.05). Among females, indoor falls made up a larger share of the total falls when compared to males (females: 65.31%; males: 60.6%). In contrast, the share of outdoor falls in men (12.57%) was higher than falls among women (8.48%). While indoor falls progressively increased with age, they represented the highest share of falls among the oldest of the old (85 years and over: 65.96%); outdoor falls were most represented among the 65–74 year olds (14.90%).

Compared to micropolitan and rural areas, indoor falls made up a higher share of total falls in metro areas (large: 64.1%; small: 65.24%). In contrast, the percentage of outdoor falls was slightly higher in micropolitan and rural areas (more than 11%) than that in metro areas (less than 10%). While 63.95% of the total falls paid by Medicare were indoor, 9.44% were outdoor. Private insurance, on the other hand, paid for 61.08% of indoor falls, and 13.98% of outdoor falls. Those living in ZIP codes with an income above $51,000 had a slightly higher share of indoor (approximately 64%) and outdoor (over 10%) falls compared to those living in ZIP codes below $40,000 (63.11%, and 8.89%). Outdoor falls were represented the most among those with a score of “0 = no chronic conditions” on the GRPCI (12.60%), while the least among those with a score of “2 = multiple chronic conditions” (6.26%).

#### Multivariable Analysis

We present the results from our multivariable analysis (multinomial logistic regression) in Table 3. In Model 1, we present the results for indoor and outdoor fall outcomes, and in Model 2 we substitute the GRPCI with the 17 chronic conditions as predictor variables in the analysis. Females (relative risk [RR] = 1.43, 95%, confidence interval [CI], 1.42–1.44), and older adults over 85 years and above (RR = 2.35, 95%, CI, 2.33–2.37) had a higher likelihood of belonging in the indoor fall visit category as opposed to the no-fall visit category. Next, older adult residence in non-core rural areas (RR = 1.25, 95%, CI, 1.22–1.29) increased the likelihood of reporting an outdoor fall as opposed to no falls. In comparison, residence in higher income (≥ $66,000) ZIP codes increased the likelihood of belonging to an indoor (RR = 1.20, 95%, CI, 1.19–1.21) as well as an outdoor fall visit (RR = 1.65, 95% CI, 1.61–1.68).

**Figure 3:**
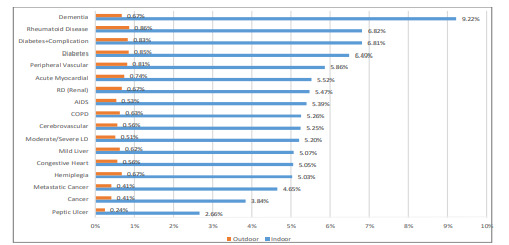
Probabilities of an indoor and outdoor fall in the presence of a chronic condition, NEDS 2017. Note: The complement of the probabilities for each chronic condition is the probability associated with no fall in the presence of the respective chronic condition.

In Model 2, we controlled for the 17 chronic conditions identified in the CCI. Both the GRPCI (Model 1) and the individual chronic conditions (Model 2) did not indicate a higher likehood of older adults belonging to any of the fall (indoor/outdoor) categories compared to the elderly reporting no falls. Nevertheless, for all 17 chronic conditions, the probabilities associated with an indoor fall in the presence of a chronic condition were consistently much higher when compared to an outdoor fall. In Figure 3, we provide the probabilities associated with an indoor fall (blue bar) in the presence (compared to an absence) of the 17 conditions. The orange bars indicate the same statistic for an outdoor fall. For instance, the probability of an indoor fall (9.22%) in the presence of dementia was followed by that of rheumatoid arthritis (6.82%) among older adults visiting the ED. In contrast these probabilities for an outdoor fall respectively were 0.67% (dementia) and 0.86% (rheumatoid arthritis).

## DISCUSSION

Using the 2017 NEDS dataset, we estimated a total of 3.5 million fall-related visits among older adults in the United States in 2017. Overall, indoor fall-related ED visits were six times higher than outdoor fall visits. We examined various factors affecting fall-related ED visits to identify and compare-contrast factors associated with indoor vs outdoor fall visits since fall prevention and mitigation strategies would be different for each type of fall. In connection, we present results to highlight implications from three perspectives – from a population health standoint for EDs working with their primary care and community care colleagues, from an ED administrative angle, and from an individual emergency clinician’s point of view.

Consistent with prior studies,^12^–^15^ our analysis found the role of intrinsic (personal) factors – gender- and age-based variations in the incidence of fall-related ED visits. Age was a significant predictor of indoor falls with the frequency of ED visits increasing more than sixfold with age across both genders. On the other hand, although an increase was seen in the frequency of ED visits with age for outdoor falls, that increase was less than twofold. Similarly, our multivariable analysis indicated that with age, the likelihood of a patient visiting the ED with an indoor fall (RR 2.35 for age>85) increased, but the same was not true for an outdoor fall. Increasing age is therefore a strong predictor of indoor fall visits. Emergency clinicians should refer older patients (>85 years) more aggressively to community resources for indoor-fall prevention programs while providing general resources for all ages for outdoor fall prevention. Furthermore, to address the needs of patients presenting with fall-related visits, ED medical directors need to account for the fact that the majority of their outdoor fall cases will be in the younger age group (Table 3) and that indoor fall cases, in all likelihood, will be evenly distributed (Table 2). This trend will be of importance when arranging services for post-fall visit discharge from the ED. At the population level, greater resources need to be dedicated for indoor-fall prevention programs for those above age 85 for the highest return on investment.

With respect to gender, women had a higher incidence of fall-related ED visits in the outdoor and indoor fall categories across all ages (except outdoor for 85 years and older). Female gender increased the probability of an indoor fall-related ED visit (as opposed to no falls) by one and a half times when compared to men, but this difference was minimal in the case of outdoor fall visits. Out of a 100 falls seen in the ED, females accounted for two thirds of the indoor fall visits. This significant gender disparity needs to be addressed when arranging for primary preventive services as well as arranging care for older adults who present to the ED with falls. Females will need greater attention in all fall prevention and mitigation programs at the individual as well as the population level. On the administrative side, greater fall-prevention resources will need to be allocated for female patients.

Additionally, compared to an outdoor fall, the probabilities of an indoor fall were higher in the presence of all 17 chronic conditions that we considered in our analysis (Figure 3). This difference was far higher for each of these chronic health conditions than the sixfold gap between the incidences of indoor and outdoor fall-related ED visits. While previous studies have primarily examined the relation between risk of falling and the presence of a particular chronic condition,^8^ our study finds robust evidence of a higher likelihood of falling in an indoor setting in the presence of this group of 17 chronic conditions. The prevalence of multiple chronic conditions among older adults in the US is not only high but is also increasing over time,^23^ rendering effective indoor falls prevention a public health priority. Thus, emergency clinicians may be able to use the presence of these particular chronic conditions to identify patients at risk of indoor falling. Use of fall precautions in patients being admitted to the hospital from the ED or being discharged home from the ED should be based on the presence/absence of these chronic health conditions.

Our results also revealed that the the cost of care for an indoor fall visit was greater than for an outdoor fall. We estimated the total charges associated with falls seen in EDs in the US were over $17 billion in 2017. Of this total, a disproportionate 34% was borne by Medicare to reimburse fall visits in the ED for older adults 85 years and over. Out of every 100 falls seen in the ED almost 90 are paid by Medicare. This was true for indoor as well as outdoor location of falls. In 2017, the estimated population of adults aged 85 and over was over six million,^22^ of which over two-thirds were women. The 85 years and over population is projected to reach 19 million in 2050.^24^ With this increase, the number of indoor and outdoor falls, and associated costs are expected to rise. Consequently, the need for effective falls prevention, especially indoor falls among women, is urgent.

In addition to the intrinsic factors, our results also identified personal- and community-level factors for fall-related ED visits. With respect to patient residence across communities (metropolitan, micropolitan, non-core), living in a metropolitan area increased the likelihood of an older adult reporting an indoor fall compared to a higher likelihood of an outdoor fall in non-core rural areas. While emergency clinicians should take note of this trend, population health and ED administrative startegic planning may similarly need appropriate tailoring in urban vs rural areas. Results from the multinomial logistic regression analyses (Table 3) also indicated a higher likelihood of indoor and outdoor fall-related visits among those in high-income ZIP codes. This finding, in all likelihood, highlights the disparity in access to resources for patients residing in low-income areas. At the population level, all in the healthcare system need to address economic disparities in access to care, specifically access to ED care for those in low-income areas. Additionally, individual emergency clinicians need to remain aware that all patients, including those from a higher income bracket, will need referal to fall prevention and mitigation care upon discharge from the ED.

In conjunction to the above, we also highlight the role of the multidisciplinary ED team comprised of emergency physicians, nurses, social workers, case managers, and counselors to help mitigate the effects of these personal (intrinsic) and socioeconomic (extrinsic) factors that may be contributing to the increasing volume of fall-related indoor/outdoor visits among our elderly. With fall-related ED visits on the rise, analysts^2^ have highlighted the potential role that EDs could play in falls-prevention, and in conjunction the need for research on types of programs administrable in EDs. The EDs are in a unique position to engage and educate the older adults about future falls prevention. In 2014, the American College of Emergency Physicians, American Geriatric Society, Emergency Nurses Association, and Society for Academic Emergency Medicine released geriatric guidelines specific for EDs that recommend screening for fall risk in EDs.^25^ Indeed, a collective assessment that includes evaluation of current level of knowledge in addition to patient’s balance, history of falls, and home evaluations is essential,^4^ especially for those 85 years and older, female, or with chronic conditions. In fact, EDs incorporating a clinical support tool, such as the Stopping Elderly Accidents, Deaths, and Injuries, in conjunction with primary care providers saw a subsequent decrease in fall-related hospitalizations^26^ and were successful in delivering high-quality care.^1^ In addition, a geriatric-friendly protocol^27^ that facilitates community service providers and/or geriatricians to collaborate with EDs for fall prevention could be beneficial.

## LIMITATIONS

While our study is the first national-level study to report evidence of heterogeneity of risk factors by fall locations of older adults across the US, this finding is subject to a few limitations. First, the NEDS dataset collects visits-level information without designating any unique identifiers to patients. Thus, we could not determine instances of multiple records for the same patient. Despite this shortcoming, NEDS is the one exception that provides robust national estimates of hospital-based ED visit characteristics using the ICD-10-CM classification as opposed to self-reported data on falls. Second, while we controlled for patient’s location as a proxy indicator for indoor/outdoor exposure, variation in fall types due to indoor and outdoor environments is an important future research direction.

Finally, while some of the ICD-10-CM codes (for example, W06-fall from bed, W14-fall from tree) were easily and clearly classifiable into an indoor (or outdoor) fall type, for others, we had to rely on evidence from the prior literature. For example, prior research^28^–^33^ indicated elderly falls on the same level from slipping, tripping and stumbling (W01) to occur predominantly at home and so we categorized this ICD-10-CM code as an indoor fall. With this method, we acknowledge that we may have misclassified any portion of the same-level geriatric falls that occurred outside.

## CONCLUSION

Older adult falls are complex, resulting from intrinsic conditions (such as chronic disease, frailty), extrinsic (environmental) factors, and/or situational activity. Emergency department encounters specific to older adult falls are associated with substantial costs, particularly to the Medicare program. Using the nationally representative 2017 NEDS dataset, we estimated a total of 3.52 million falls among older adults seen in the ED and found that risk factors of these falls varied by fall indoor/outdoor locations. When compared to older adult reporting no falls, women, those over 85 years, those with chronic conditions, and those from metropolitan areas had a higher likelihood of reporting indoor falls in the ED. In conjunction, we highlighted implications from three perspectives: a population health standoint for EDs working with their primary care and community care colleagues, from an ED administrative vantage point; and from an individual emergency clinician’s point of view. Findings of our study are of salience in interpreting falls in EDs across the US. Indeed, reducing fall-related ED visits and, in turn, ED-based falls prevention programs are a public health priority.

## Figures and Tables

**Table 1:** List of variables included in the bivariate (chi-square) and multivariable (logistic regression) analyses.

Variable	Indicator	Description		
*Outcome variable:*				
Fall event of older adults (≥ 65 years)	Bivariate analysis: Fall-related visits disaggregated by fall location: indoor, outdoor, and other (N = 6,670,508)	Indoors/outdoors falls definition in Kelsey et al (2010) applied to ED visits with ICD-10-CM diagnoses codes (W codes) for those 65 years and older (additional details with the list of W codes as indicated below).		
		ICD-10-CM W codes (W00-W19) for Falls		
	Multivariable analysis: Fall-related visits aggregated for indoor and outdoor fall locations (N = 6,455,630)	Indoor: W010XXA W0110XA W01110A W01111A W01118A W01119A W01190A W01198A W03XXXA W04XXXA W050XXA W06XXXA W07XXXA W08XXXA W16211A W16212A W16221A W16222A W1811XA W1812XA W182XXA W1830XA W1831XA W1839XA	Outdoor: W000XXA W001XXA W002XXA W009XXA W051XXA W052XXA W090XXA W091XXA W092XXA W098XXA W100XXA W101XXA W102XXA W108XXA W109XXA W11XXXA W12XXXA W130XXA W131XXA W132XXA W133XXA W134XXA W138XXA W139XXA W14XXXA W15XXXA W16011A W16012A W16021A W16022A W16031A W16032A W16111A W16112A W16121A W16122A W16131A W16132A	Other: W1800XA W1801XA W1802XA W1809XA W19XXXA
*Predictor variables*				
Patient characteristics (intrinsic/personal)				
Gender^1^	(Female/Male)	Binary categorical variable.		
Age	Age groups	Categorical variable with three levels: 65–74 years; 75–84 years; 85 years and above.		
Health	Individual chronic conditions	Chronic conditions identified in the computation of the Charlson Comorbidity Index and Grouped Charlson Comorbidity Index and as listed below. Myocardial infarction, congestive heart failure, peripheral vascular disease, cerebrovascular disease, dementia, chronic obstructive pulmonary disease, ulcer, liver disease, diabetes, diabetes with complications, rheumatoid disease, moderate to severe liver disease, hemiplegia, renal disease, cancer, metastatic cancer, and acquired immunodeficiency syndrome.		
Personal- and community-level enabling resources				
Insurance	Primary payer	Categorical variable with four levels: Medicare; Medicaid and other payor; private insurance; uninsured (including self-pay and no charge).		
Location	Rurality/urbanity of patients’ ZIP codes	Categorical variable with four levels: large metropolitan areas; small metropolitan areas; micropolitan areas; non-core areas (rural), using classification provided in NEDS.		
Income	Median household income of patients’ ZIP codes	Categorical variable with four levels: less than 40,000; 40,000–50,999; 51,000–65,999; 66,000 and above.		

1 The gender variable corresponds to the NEDS data element “Female,” which is an indicator of gender.17 It therefore includes the binary male/female categories instead of the non-binary gender identity categories.

*N*, weighted observations; *ICD-10-CM*, International Classification of Diseases, 10th Revision, Clinical Modification; *NEDS*, Nationwide Emergency Department Sample.

**Table 2:** Key sociodemographic characteristics of older adults (≥ 65 years) reporting falls in the ED, NEDS 2017.^a^*

Variables^b^	Total older adult ED visits % (SE) [CI] (n=6,670,508) (N=28,988,938)	Falls, % (SE) [CI]		

		Total falls^c^ (n=812,400) (N=3,529,861)	Indoor falls (n=519,145) (N=2,247,417)	Outdoor falls (n=78,377) (N=349,632)
	Predictor categories add up to 100% column-wise	Predictor categories add up to 100% column-wise	Row-wise (Indoor + Outdoor + Other [not shown in the table]) adds up to 100%	
Total				
Gender	6,670,129†	812,370†		0.00††
Male	43.05 (0.02) [43.01, 43.08]	34.80 (0.05) [34.70,34.91]	60.60 (0.09) [60.41, 60.78]	12.57 (0.06) [12.44, 12.70]
Female	56.95 (0.02) [56.92, 56.99]	65.20 (0.05) [65.09,65.30]	65.31 (0.07) [65.18, 65.44]	8.48 (0.04) [8.41, 8.56]
Age group	6,670,508†	812,400†		0.00††
65–74 years	45.31 (0.02) [45.27, 45.35]	33.30 (0.05) [33.19,33.40]	60.91 (0.096) [60.72, 61.1]	14.90 (0.07) [14.76, 15.04]
75–84 years	33.46 (0.02) [33.43, 33.5]	34.22 (0.05) [34.12, 34.33]	64.18 (0.093) [64.0, 64.36]	9.57 (0.06) [9.46, 9.68]
85 years and over	21.23 (0.02) [21.19, 21.26]	32.48 (0.05) [32.37, 32.58]	65.96 (0.095) [65.77, 66.14]	5.14 (0.04) [5.05, 5.23]
Payer	6,656,643†	810,595†		0.00††
Medicare	87.26 (0.01) [87.23, 87.28]	89.50 (0.03) [89.43, 89.57]	63.95 (0.06) [63.84, 64.07]	9.44 (0.035) [9.37, 9.51]
Medicaid and other	3.30 (0.007) [3.29, 3.31]	2.70 (0.02) [2.66, 2.74]	61.77 (0.34) [61.10, 62.43]	13.16 (0.24) [12.70, 13.64]
Private insurance	8.14 (0.01) [8.12, 8.17]	6.83 (0.03) [6.78, 6.89]	61.08 (0.21) [60.67, 61.50]	13.98 (0.15) [13.69, 14.29]
Self-pay/No pay	1.30 (0.004) [1.29, 1.31]	0.97 (0.01) [0.95, 0.99]	61.54 (0.56) [60.44, 62.63]	13.18 (0.39) [12.43, 13.97]
Location	6,651,198†	810,272†		0.00††
Large metro areas	48.5 (0.008) [48.48, 48.51]	48.91 (0.02) [48.86, 48.96]	64.10 (0.08) [63.95, 64.25]	9.60 (0.05) [9.51, 9.70]
Small metro areas	32.5 (0.01) [32.48, 32.52]	33.27 (0.03) [33.22, 33.32]	65.24 (0.09) [65.06, 65.43]	9.54 (0.06) [9.43, 9.66]
Micropolitan areas	10.95 (0.008) [10.93, 10.97]	10.20 (0.02) [10.16, 10.25]	60.88 (0.18) [60.52, 61.23]	11.18 (0.12) [10.95, 11.41]
Non-core areas	8.05 (0.007) [8.04, 8.07]	7.62 (0.02) [7.58, 7.66]	57.80 (0.21) [57.38, 58.22]	11.66 (0.14) [11.39, 11.93]
Income Level	6,559,393†	799,987†		0.00††
$1–$39,000	28.09 (0.02) [28.06, 28.12]	24.79 (0.05) [24.70, 24.88]	63.11 (0.11) [62.89, 63.33]	8.89 (0.07) [8.76, 9.02]
40,000–$50,999	27.01 (0.02) [26.97, 27.04]	26.66 (0.05) [26.56, 26.76]	63.59 (0.11) [63.38, 63.8]	9.86 (0.07) [9.73, 10.0]
51,000–$65,999	23.96 (0.02) [23.93, 24.0]	25.03 (0.05) [24.94, 25.13]	64.23 (0.11) [64.01, 64.44]	10.26 (0.07) [10.12, 10.40]
66,000 or more	20.94 (0.01) [20.91, 20.97]	23.52 (0.04) [23.44, 23.61]	63.81 (0.11) [63.59, 64.03]	10.55 (0.07) [10.40, 10.69]
Grouped Charlson Comorbidity Index (GRPCI)	6,670,508†	812,400†		0.00††
0	40.19 (0.02) [40.16, 40.23]	48.75 (0.06) [48.64, 48.86]	63.93 (0.08) [63.78, 64.09]	12.60 (0.05) [12.49, 12.70]
1	23.73 (0.02) [23.69, 23.76]	25.02 (0.05) [24.92, 25.11]	63.81 (0.11) [63.59, 64.02]	8.48 (0.06) [8.36, 8.61]
2	36.08 (0.02) [36.04, 36.12]	26.23 (0.05) [26.14, 26.33]	63.04 (0.11) [62.83, 63.25	6.26 (0.05) [6.15, 6.37]

* The instructions provided by the Agency for Healthcare Research and Quality: Healthcare Cost and Utilization Project (HCUP) directed the statistical procedure we used to generate the national estimates and descriptive statistics (confidence intervals and standard errors) for falls by each falls category as well as by predictor variables.

a We used the sampling weights provided by the HCUP NEDS dataset to generalize the estimates to the US civilian, noninstitutionalized adult population.

b Missing value for predictors variables: The maximum was 1.5% for income.

c Total unweighted fall-related visits (N = 812,400) include three fall location categories: i) indoor (519,145); ii) outdoor (78,377); and iii) other (N = 214,878).

† Unweighted observations (n with no missing values) for each predictor variables;

†† χ2 P values.

*SE*, standard error; *CI*, confidence interval; *n*, unweighted observations with no missing values, *N*, weighted observations, *NEDS*, Nationwide Emergency Department Sample.

**Table 3:** Multivariable multinomial logistic regression analysis (N = 27,522,770 (weighted)): Predictors of indoor falls (0 = no falls; 1 = indoor; 2 = outdoor) of older adults (≥ 65 years), NEDS 2017.

Population >=65 years	MODEL 1 RR, [CI] [Base category: no falls]	P-value	MODEL 2 RR, [CI] [Base category: no falls]	P-value
Indoor falls				
Gender				
Male	Ref		Ref	
Female	1.46 [1.45–1.46]	0.000	1.43 [1.42–1.44]	0.000
Age group				
65–74 years	Ref		Ref	
75–84 years	1.55 [1.54–1.56]	0.000	1.51 [1.49–1.52]	0.000
85 years and over	2.53 [2.51–2.55]	0.000	2.35 [2.33–2.37]	0.000
Location				
Large metro areas	Ref		Ref	
Small metro areas	1.06 [1.05–1.06]	0.000	1.06 [1.06–1.07]	0.000
Micropolitan areas	0.90 [0.88–0.91]	0.000	0.91 [0.90–0.92]	0.000
Non–core areas	0.87 [0.86–0.88]	0.000	0.88 [0.87–0.89]	0.000
Payer				
Medicare	Ref		Ref	
Medicaid and other	0.91 [0.89–0.93]	0.000	0.91 [0.89–0.92]	0.000
Private insurance	0.87 [0.85–0.88]	0.000	0.86 [0.85–0.87]	0.000
Self-pay/No pay	0.73 [0.71–0.76]	0.000	0.72 [0.70–0.75]	0.000
Income level				
$1–$39,000	Ref		Ref	
$40,000–$50,999	1.09 [1.08–1.10]	0.000	1.10 [1.09–1.10]	0.000
$51,000–$65,999	1.13 [1.12–1.14]	0.000	1.14 [1.13–1.15]	0.000
$66,000 or more	1.20 [1.19–1.21]	0.000	1.20 [1.19–1.21]	0.000
Grouped Charlson Comorbidity Index (GRPCI)				
0	Ref			
1	0.81 [0.80–0.81]	0.000	-	-
2	0.52 [0.51–0.52]	0.000	-	-
Outdoor falls				
Gender				
Male	Ref		Ref	
Female	0.96 [0.95–0.97]	0.000	0.96 [0.94–0.97]	0.000
Age group				
65–74 years	Ref		Ref	
75–84 years	1.00 [0.98–1.01]	0.709	1.00 [0.99–1.02]	0.709
85 years and over	0.89 [0.87–0.91]	0.000	0.90 [0.88–0.92]	0.000
Location				
Large metro areas	Ref		Ref	
Small metro areas	1.07 [1.05–1.08]	0.000	1.07 [1.05–1.09]	0.000
Micropolitan areas	1.16 [1.13–1.19]	0.000	1.17 [1.14–1.20]	0.000
Non-core areas	1.25 [1.21–1.28]	0.000	1.25 [1.22–1.29]	0.000
Payer				
Medicare	Ref		Ref	
Medicaid and other	1.07 [1.03–1.12]	0.002	1.06 [1.02–1.11]	0.002
Private insurance	1.04 [1.02–1.07]	0.004	1.04 [1.01–1.06]	0.004
Self-pay/no pay	0.86 [0.81–0.92]	0.000	0.85 [0.80–0.91]	0.000
Income level				
$1–$39,000	Ref		Ref	
$40,000–$50,999	1.24 [1.21–1.26]	0.000	1.24 [1.21–1.27]	0.000
$51,000–$65,999	1.43 [1.39–1.46]	0.000	1.43 [1.40–1.47]	0.000
$66,000 or more	1.63 [1.59–1.66]	0.000	1.65 [1.61–1.68]	0.000
17 Chronic conditions controlled	No		Yes	

* Missing values were about 3% of the sample.

*RR*, relative risk ratio; *CI*, confidence interval; *N*, observations; *NEDS*, Nationwide Emergency Department Sample.
